# Saline nasal irrigation for acute sinusitis (SNIFS II): a randomised controlled pilot trial with nested process evaluation

**DOI:** 10.3399/BJGPO.2024.0307

**Published:** 2025-08-27

**Authors:** Roderick P Venekamp, Ben Ainsworth, Tammy Thomas, Beth Stuart, Joanna Slodkowska-Barabasz, Fiona Mowbray, Christopher C Butler, Nick Francis, Samantha Richards-Hall, Anthony Harnden, Alastair D Hay, Claire Hopkins, Michael Moore, Lucy Yardley, Theo JM Verheij, Shihua Zhu, Paul Little

**Affiliations:** 1 Department of General Practice and Nursing Science, Julius Center for Health Sciences and Primary Care, University Medical Center Utrecht, Utrecht University, Utrecht, The Netherlands; 2 School of Psychology, University of Southampton, Southampton, UK; 3 Primary Care Research Centre, Primary Care & Population Sciences Unit, Aldermoor Health Centre, Faculty of Medicine, University of Southampton, Southampton, UK; 4 Wolfson Institute of Population Health, Queen Mary University of London, London, UK; 5 Centre for Clinical and Community Applications of Health Psychology, University of Southampton, Southampton, UK; 6 Nuffield Department of Primary Care Health Sciences, University of Oxford, Oxford, UK; 7 Primary Care Research Centre, Primary Care Population Sciences and Medical Education Unit, University of Southampton, Southampton, UK; 8 University of Bristol, Centre for Academic Primary Care, Bristol Medical School, Population Health Sciences, Bristol, UK; 9 Department of Rhinology and Skull Base Surgery, Guy’s and St Thomas’ Hospital NHS Foundation Trust, London, UK; 10 School of Psychological Science, University of Bristol, Bristol, UK; 11 Health Economics Department, Primary Care & Population Sciences Unit, Aldermoor Health Centre, Faculty of Medicine, University of Southampton, Southampton, UK

**Keywords:** acute sinusitis, sinusitis, primary health care, saline nasal irrigation, nasal lavage

## Abstract

**Background:**

Despite having marginal beneficial effects, antibiotics are routinely prescribed in adults with acute sinusitis. Alternative interventions for this common condition are urgently needed.

**Aim:**

To assess the feasibility and acceptability of saline nasal irrigation for acute sinusitis.

**Design & setting:**

Randomised controlled pilot trial with nested process evaluation in 24 English general practices between October 2019 and May 2021.

**Method:**

Participants were randomised to advice to high-volume hypertonic saline nasal irrigation with a delayed antibiotic prescription or usual care. Feasibility outcomes included recruitment and follow-up rates, adherence, and acceptability of the intervention.

**Results:**

Of those invited, *n* = 81/107 (76%) consented and were randomised (42 intervention, 39 usual care). Two participants were excluded owing to ineligibility. Antibiotic prescribing strategies were recorded at baseline for *n* = 79/79 (100%), with no or delayed antibiotics prescribed in 60% (*n* = 24/40) of the saline group versus 38% (*n* = 15/39) of the usual care group. At follow-up, 80% (*n* = 63/79) of participants recorded whether they consumed antibiotics or not. Among those from the intervention group who returned a symptom diary, 96% (*n* = 22/23) and 65% (*n* = 15/23) reported using saline nasal irrigation during the first and second week, respectively. Semi-structured interviews with 16 participants revealed that most were positive about trial participation and viewed saline nasal irrigation as acceptable, noting it as an alternative to antibiotics.

**Conclusion:**

Saline nasal irrigation is deemed acceptable for adults with acute sinusitis and a trial of such intervention is feasible. A large trial is warranted to assess the effectiveness of this intervention for this common condition.

## How this fits in

Despite having only marginal beneficial effects, antibiotics are routinely prescribed in adults with acute sinusitis. Alternative interventions to effectively relieve symptoms and reduce the reliance on antibiotics for this very common condition are currently lacking. Our randomised controlled pilot trial, with nested process evaluation in UK primary care, showed that a brief intervention to advise high-volume hypertonic saline nasal irrigation with a delayed antibiotic prescription in adults with acute sinusitis is deemed acceptable and a trial of such intervention in primary care is feasible. A large trial is therefore warranted to assess the clinical and cost-effectiveness of this intervention for this common condition.

## Introduction

Acute sinusitis is among the most common infections in adults.^1^ Although mostly self-limiting and incurring a very low risk of serious complications,^2^ associated costs are high owing to frequent primary care visits, antibiotic prescriptions, over the counter (OTC) medication use, and lost productivity.^3^ The annual costs of antibiotic prescribing for the condition has been estimated at around 10 million in the UK, and 2.4 billion dollars in the US.^4^

Despite the limited benefit from antibiotics, antibiotic prescribing rates remain very high.^5^ In fact, sinusitis accounts for the acute respiratory tract infection (ARTI) with the highest percentage of patients receiving antibiotics.^5^ It is also among conditions with the highest antibiotic overprescribing rates in adults,^6^ thereby exposing patients to avoidable side effects^7^ and the population to emerging antimicrobial resistance.^8^ With antimicrobial resistance posing a serious threat to public health globally, alternative interventions to effectively relieve symptoms and reduce the reliance on antibiotics for this common condition are urgently needed.

Saline nasal irrigation may potentially be a low-cost, low-risk alternative. The European Position Paper on Rhinosinusitis and Nasal Polyps (EPOS) 2020 suggests that this might be a suitable option to relieve ARTI symptoms, but acknowledges that the evidence is poor.^9^ Similarly, a Cochrane review on saline nasal irrigation for acute upper respiratory tract infections concludes that the trials conducted so far were small and had a high risk of bias.^10^ In addition, a Cochrane review of saline irrigation for chronic sinusitis concluded that there may be some benefit from daily, large-volume saline irrigation with a hypertonic solution compared with placebo.^11^ More recently a brief intervention providing simple instructions on how to do high-volume nasal irrigation using a neti pot was trialled in recurrent and chronic sinusitis.^12^ Compared with previous trials that used more intensive individual coaching in how to do nasal irrigation, this pragmatic trial demonstrated smaller — but still important — symptomatic benefits.^13,14^ The intervention was very simple and most participants were still using nasal irrigation 6 months later. They reported reduced intention to consult a doctor in future episodes, and were also less likely to use OTC medication. This indicates that well-designed, low-cost, and scalable interventions may be feasible, acceptable, and potentially cost-effective to support patients with acute sinusitis without placing additional demands on healthcare resources. Qualitative work highlighted that targeted advice about overcoming initial problems with irrigation techniques could potentially have helped increase patients’ behavioural engagement.

Based on these considerations, we aimed to conduct a randomised controlled pilot trial with nested process evaluation in UK primary care, to assess the feasibility and acceptability of this relatively simple intervention for adults with acute sinusitis.

## Method

### Design

Between October 2019 and May 2021**,** we conducted an open-label, individually randomised (1:1) controlled pilot trial with nested process evaluation in 24 English general practices.

### Participants

Eligible participants were patients aged ≥18 years attending primary care with acute sinusitis defined as having sinus discomfort, and at least two of the following symptoms: patient-reported nasal obstruction, patient-reported purulent nasal discharge, or pus seen in the nasal cavity on inspection by the clinician. Patients were excluded who were unable to complete outcomes owing to dementia or severe uncontrolled mental illness; who had terminal illness; who were pregnant or breastfeeding; and who had head or neck cancer, cystic fibrosis, other nasal disorders including polyps, or immunodeficiencies caused by, for example, HIV or immune-suppressive treatment.

Participating GPs informed potentially eligible participants about the study verbally and via a patient information leaflet. Patients who provided written consent to the study were randomised via a trial randomisation website to ensure concealed study treatment assignment to either intervention or control group, using a computer-generated sequence list with stratification, according to the prior duration of illness (<7 days or ≥7 days).

### Intervention and comparator

In addition to advice about the use of analgesics as per usual care, participants in the intervention group were given both verbal and written advice (the *Rinse It Out!* booklet; Supplementary File 1) and a link to a video clip (http://www.youtube.com/watch?v=zgvoxkGYSU4) demonstrating how to perform irrigation. The booklet and video were developed and optimised using the person-based approach^15^ to ensure it was as acceptable, engaging, and as effective as possible. In this iterative process, 16 adults who had experienced acute sinusitis were interviewed and provided detailed feedback about the advice in ‘think-aloud interviews’. The booklet and video advice were optimised after each interview until no further negative feedback was obtained.

Participants were asked to irrigate the nose (150 ml through each nostril) using a SinuCleanse 19 nasal cup (neti pot) daily for up to 21 days or until symptoms had settled. Patients were instructed to make their own buffered saline irrigation solution every 1 to 2 days comprising: one heaped teaspoon salt, one half teaspoon baking soda, and 1 pint (568 ml) tap water.^12,13^ Participants in the intervention group were also offered a delayed antibiotic prescription to be filled if symptoms were getting significantly worse or had not started to settle a little after a further week, as in our previous trials.^16^

The control group received usual care. Since more than 90% of patients currently receive antibiotics for acute sinusitis,^5^ the nearest approximation to usual care is a prescription for immediate antibiotics, combined with advice about the use of analgesics. Although the particular antibiotics used was not the focus of this investigation, GPs were advised on the use of penicillin V 500 mg QDS or alternatively amoxicillin 500 mg TDS for 1 week or clarithromycin 500 mg BD also for 1 week in case of penicillin allergy, as per the Public Health England (PHE) guidance for primary care.^17^

Taking a pragmatic approach, all further management decisions during follow-up, such as further medication or referral, were at the discretion of the doctor according to the normal practice of that doctor.

### Data collection

Participants kept a diary of symptoms (Supplementary File 2), including 11 symptom variables assessed on 7-point Likert scales,^18,19^ and daily activities for up to 4 weeks after inclusion or until symptoms had settled. The format of the diary items has been developed in a variety of ARTIs, including sinusitis, and been shown to have construct and criterion validity, and also sensitivity to change.^20–22^

If no diary was received, a brief questionnaire was sent to capture the key outcomes of interest. We reached out via a phonecall and/or text message to those not having returned the brief questionnaire.

### Outcomes

The feasibility outcomes of interest were recruitment rate, proportion of eligible patients who accepted randomisation, treatment adherence in the intervention group, and proportion in which the main clinical outcome data were captured in each group.

The main clinical outcome of interest was patient-reported antibiotic consumption during 4-weeks follow-up. Secondary clinical outcomes included the antibiotic prescription strategy used by the GP; the duration of moderately bad illness,^18,19^ which score has been developed to have content validity by incorporating items that not only take account of proposed diagnostic criteria for bacterial sinusitis,^23^ but also physicians’ perceptions of the important clinical features;^24^ symptom score on days 2–4; and primary care re-consultation with new, non-resolving, or worsening illness within 4 weeks of the index consultation documented from medical records.^25^

### Nested process evaluation

In a process evaluation alongside the trial, we explored a range of patients’ views and experiences on trial participation by conducting semi-structured in-depth interviews with participants from both groups. To ensure key emerging issues were captured and participants’ views were represented, we used a flexible and subtle realist approach. Participants were invited to speak freely on topics related to the intervention, which allowed for the exploration of their engagement with the intervention. Participants described how the intervention was delivered, how they used it, how they found its different components (that is, video and booklet with instructions), and what they perceived to be advantages and disadvantages of this intervention. A purposive sampling approach — taking into account factors such as sex and rural or urban settings — was designed to invite views of a broad range of patients. All interviews were audio-recorded and transcribed verbatim.

### Sample size considerations

To estimate follow-up rates between 65% and 80% with accompanying 95% confidence intervals (CIs) of ± 15% in each group, 41 participants per group were needed. This sample size would detect antibiotic consumption and prescription of 65% or less in the intervention group with 95% CI of ±15%. For the process evaluation, an estimated 15–30 interviews was considered adequate to represent the views of a range of patients following trial participation and to reach data sufficiency.

### Statistical analysis

All analyses were performed according to the intention-to-treat analysis principle. Baseline characteristics by trial group were described descriptively. For all feasibility and clinical outcomes of interest, results were presented descriptively. Continuous outcomes were expressed as means with standard deviations (SD) or median with interquartile ranges (IQR). To control for imbalances in baseline symptom severity score and prior duration of illness, we performed regression modelling for clinical outcomes. All quantitative data were analysed using Stata (version 16.0).

### Analysis of process evaluation

For the process evaluation, we followed the stages of Braun and Clarke’s thematic analysis,^26^ supported by NVivo (version 12.0.0). Two researchers (JSB, FM) independently coded the first three interview transcripts line by line, after which they discussed the initial codes and identified potential categories and subcategories. The interview guides were refined throughout the iterative process of data collection and analysis. Subsequent coding was done by JSB. To further ensure rigour and trustworthiness at each stage of the analysis process, these stages of analysis were discussed and adapted, if necessary, within the research team.

## Results

### Study participants and feasibility outcomes

**Figure 1.**
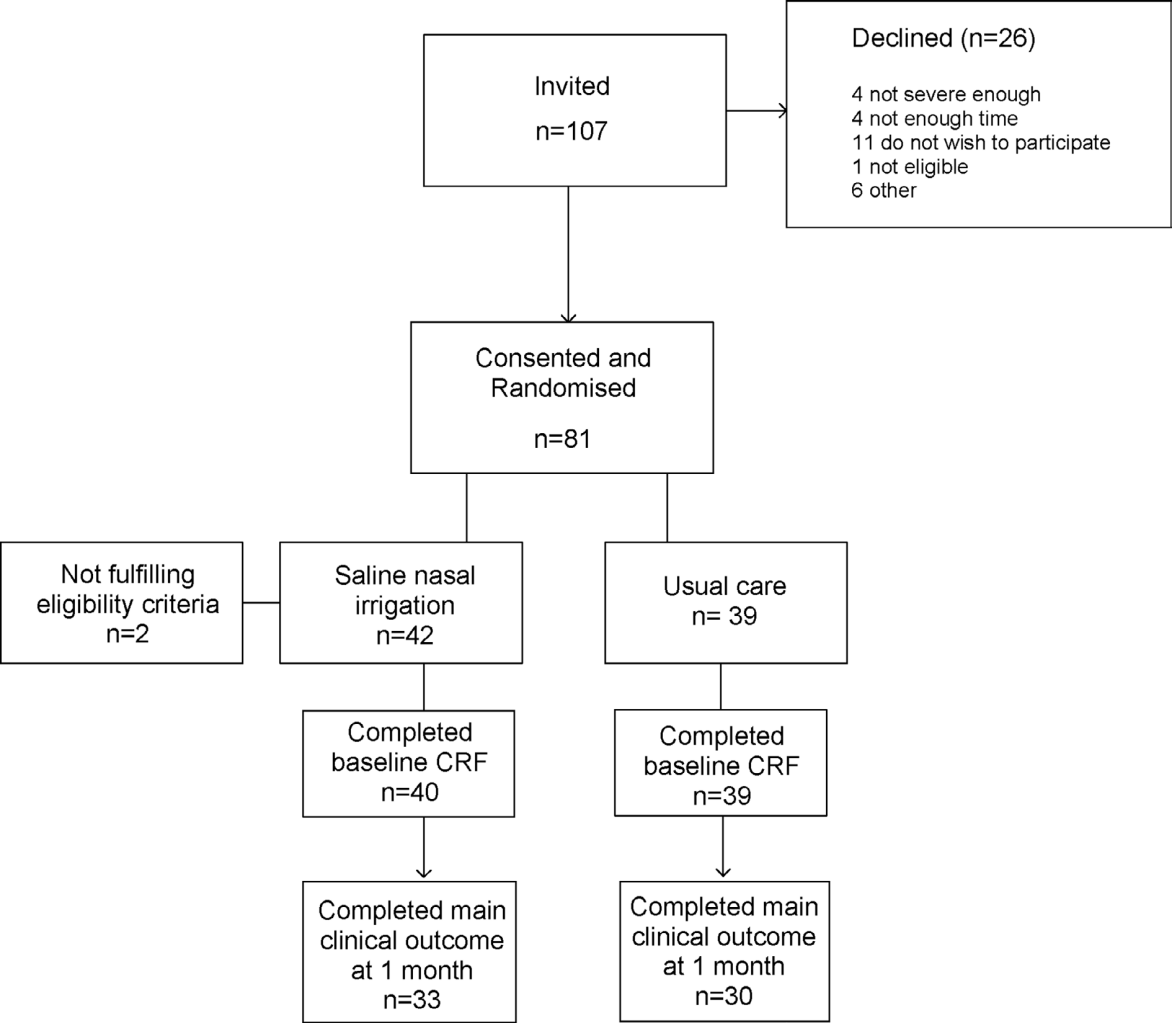
Flowchart study participants. CRF = case report form

Figure 1 illustrates the flow of participants through the trial. A total of 107 adults with acute sinusitis were invited to take part. Of those, 81 (76%) consented and were randomised (42 saline nasal irrigation, 39 usual care; Figure 1). Two participants were excluded owing to ineligibility, leaving 79 suitable for further trial participation (40 saline nasal irrigation, 39 usual care).

Table 1 summarises the baseline characteristics of participants. In the saline nasal irrigation group there were slightly more females and participants were slightly older, and by chance had a much longer illness duration before randomisation. Antibiotic prescription strategy was captured in *n* = 79/79 (100%), with no or delayed antibiotics prescribed in 60% (*n* = 24/40) of the saline group versus 38% (*n* = 15/39) of the usual care group. Antibiotic consumption data were captured in 80% (*n* = 63/79). Among those from the intervention group who returned a symptom diary, 96% (*n* = 22/23) and 65% (*n* = 15/23) reported using saline nasal irrigation at least once during the first and second week, respectively.

**Table 1.** Baseline characteristics

	Saline nasal irrigation (*n* = 40)	Usual care (*n* = 39)	Total (*n* = 79)
Mean age, years (SD)	49.0 (15.6)	46.0 (13.2)	47.6 (14.4)
Sex, female (%)	33 (82.5)	30 (76.9)	63 (79.7)
Mean symptom severity score (SD)	1.8 (0.57)	2.0 (0.50)	1.9 (0.54)
Prior duration of illness in days, median (IQR)	14 (7, 21)	11 (7, 14)	14 (7, 21)
History of repeated illness (%)	14 (35.0)	13 (33.3)	27 (34.2)
Antibiotic prescribing at baseline (%)			
Immediate antibiotics	16 (40.0)	24 (61.5)	40 (50.6)
Delayed antibiotics	7 (17.5)	3 (7.7)	10 (12.7)
No antibiotics	17 (42.5)	12 (30.8)	29 (36.7)

IQR = interquartile range. SD = standard deviation

### Clinical outcomes

The clinical outcomes of interests are summarised in Table 2.

**Table 2.** Clinical outcomes of interest

	Saline nasal irrigation (*n* = 36)	Usual care (*n* = 34)	Total (*n* = 70)
Symptom score on days 2–4, mean (SD)	2.7 (1.25)	3.0 (1.10)	2.8 (1.18)
Duration of illness, median (IQR)	8 (4,12)	5 (3,11)	6.5 (4,12)
	**Saline nasal irrigation (*n* = 33)**	**Usual care (*n* = 30)**	**Total (*n* = 63)**
Reporting taking antibiotics during 4-weeks follow-up (%)	19 (57.6)	12 (40.0)	31 (49.2)
	**Saline nasal irrigation (*n* = 26)**	**Usual care (*n* = 22)**	**Total (*n* = 48)**
Reconsultation in primary care	8 (30.7)	11 (50.0)	19 (39.6)

IQR = interquartile range. SD = standard deviation

Antibiotic use during 4-weeks follow-up was reported in 58% (*n* = 19/33) of the saline group versus 40% (*n* = 12/30) of the usual care group and did not statistically significantly differ between groups in adjusted analysis (adjusted odds ratio: 0.81, 95% confidence interval [CI] = 0.23 to 2.87).

The mean symptom score on days 2–4 was 2.7 (SD 1.3) in the saline nasal irrigation group and 3.0 (SD 1.1) in the usual care group, whereas the median illness duration post-randomisation was 8 (IQR 4,12) versus 5 days (IQR 3,11), respectively. In adjusted analyses, we found a small non-significant difference in symptom severity in the saline group, and no evidence of a difference in illness duration between groups (adjusted mean difference: 0.18, 95% CI = -0.44 to 0.81 and hazard ratio: 1.01, 95% CI = 0.57 to 1.80, respectively).

Primary care reconsultations with new, non-resolving, or worsening illness within 4 weeks occurred in 31% of the saline group versus 50% of the usual care group (adjusted odds ratio: 1.64, 95% CI = 0.47 to 5.74).

### Process evaluation

In total, 16 interviews were conducted; six in February and March 2020 and 10 between April and June 2021. The mean duration of the interviews was 14 minutes (range 6–26 minutes). Participants were predominantly female (11 female, five male), and the age ranged from 31–70 years.

The following three main themes were identified: (1) Experiences of doing saline nasal irrigation; (2) Nasal irrigation as an alternative to antibiotics and other medications; (3) Experiences of the study materials and trial procedure. A description of themes is below, and illustrative quotes have been provided in Table 3.

**Table 3.** Illustrative quotes from interviews with participants in the intervention group

Theme 1: Experiences of doing saline nasal irrigation	
Quote 1. Participant 9, female, 61 years old	’*Very uncomfortable initially, as it goes up the nose. It’s like having water shooting up your nose when you jump into a swimming pool … it certainly didn’t stop me wanting to try.*’
Quote 2. Participant 10, female, 31 years old	’*So where it’s salt, and it’s got like, um, bicarbonate of soda in it as well, I expected it to really hurt, and it didn’t. It’s a really weird sensation, but honestly, oh, it’s better than I thought it was gonna be.’*
Quote 3. Participant 12, male, 61 years old	*‘The instructions were very, very clear and straightforward and helpful. Yeah, I mean, I got it right the first time, I think. … Pictures were helpful, just to see, you know, the position, how, how far you had to sort of bend. Basically everything that the booklet said did, did happen, so, as I said, quite accurate and very detailed.*’
Quote 4. Participant 14, female, 51 years old	’*You could tell, basically, it, it wasn’t going the right way, you know, that my head wasn’t forward enough. I got better at it* [laughs]*.’*
**Theme 2: Nasal irrigation as an alternative to antibiotics and other medications**	
Quote 5. Participant 14, female, 51 years old	*‘As I say, it, it eased it quite a lot, and the shifting was like a decent sensation to know that actually, yeah, there’s something there and it’s moving. Um, and, yeah, it, it definitely sort of like cleared my head.*’
Quote 6. Participant 9, female, 61 years old	*‘It was easy to make up, it was easy to do and, to be honest, if that solved all my sinusitis problems, I wouldn’t have any problem keeping doing that permanently. I have thought I might do it once or twice a week ongoing, to see if it stops me getting sinusitis.’*
Quote 7. Participant 4, male, 57 years old	*‘Yeah, I’d recommend the whole process. It, it seems to have the right effect, it, the key thing for me is that it doesn’t require any medication.’*
Quote 8. Participant 9, female, 61 years old	*‘I just think it’s a great idea for somebody to be thinking how to treat sinusitis because there doesn’t seem to be an awful lot to be done normally, other than antibiotics or steroids. Which is horrid when you’ve had it for loads of years.’*
**Theme 3: Experiences of the study materials and trial procedure**	
Quote 9. Participant 3, female, 60 years old	*‘The, the diary was easy to answer, you know, very clear, and concise with it, so it didn’t take a great deal of your time up.’*
Quote 10. Participant 14, female, 51 years old	*‘It was good. It was really concise, easy to understand, easy to read. I read it through, probably referred to it maybe once or twice.’*

#### Theme 1: Experiences of doing saline nasal irrigation

Overall, participants had positive experiences of doing nasal irrigation. All participants in the intervention group said that doing nasal irrigation felt unpleasant at first. Nine out of 10 participants from the intervention group said that initial unpleasant sensation associated with pouring water through their nose did not stop them from continuing nasal irrigation (Quote 1, Table 3). Some people had initial concerns before doing irrigation but found it to be more positive than expected (Quote 2, Table 3).

Most participants learnt how to correctly do nasal irrigation after one or two attempts, and found it easy to prepare the solution. Participants found nasal irrigation instructions in the booklet and the video demonstration to be very comprehensive and easy to follow, including those for whom English was not their first language. Most participants felt confident to do nasal irrigation after reading the booklet and instructions once (Quote 3, Table 3). Participants found the instructional photos useful for some aspects such as correctly tilting their head during irrigation (Quote 4, Table 3).

#### Theme 2: Nasal irrigation as an alternative to antibiotics and other medications

Most patients in the intervention group said that doing nasal irrigation helped their sinusitis symptoms, unblocking their nose and relieving pain (Quote 5, Table 3). Many participants were happy to continue doing nasal irrigation even when their symptoms subsided (Quote 6, Table 3).

Some patients in the intervention group were particularly keen on trying nasal irrigation as an alternative to taking medications, especially antibiotics, to treat sinusitis (Quote 7, Table 3). They planned to continue doing nasal irrigation to avoid acute sinusitis attacks in the future and consequently to avoid taking antibiotics (Quote 8, Table 3).

#### Theme 3: Experiences of the study materials and trial procedure

Participants described the experience of taking part in the study as positive, it was clear from the start what they were asked to do; the documentation was perceived as comprehensive (Quote 9, Table 3), and the intervention was acceptable, easy to read and understand (Quote 10, Table 3).

## Discussion

### Summary

Our randomised controlled pilot trial in UK primary care showed that (i) the majority of invited eligible adult patients with acute sinusitis were willing to be randomised to a trial of advice to high-volume hypertonic saline nasal irrigation with a delayed antibiotic prescription versus usual care, (ii) adherence to the intervention was high with no contamination in the usual care group, and (iii) trial procedures, including clinical outcome data collection, appear feasible. Semi-structured interviews with trial participants revealed that most were positive about trial participation and viewed saline nasal irrigation as acceptable, noting it as an alternative to antibiotics.

### Strengths and limitations

Our pilot trial did meet the pre-defined sample size and is therefore sufficiently robust to provide reliable estimates of follow-up rates and feasibility. The main clinical outcome of interest, patient-reported antibiotic consumption was collected in 80% of participants, which is at the upper limit of our pre-defined estimation. Also, the nested process evaluation reached data sufficiency and did represent the views of a broad range of trial participants, although despite a purposive recruitment strategy, the interviews were predominantly conducted with female patients.

Notably, while the proportion of patients not prescribed immediate antibiotics was higher in the intervention group (60%), a smaller but still noted proportion (38%) were also not prescribed immediate antibiotics in usual care. This reflected our clinician sample’s reluctance to provide antibiotics universally in line with current National Institute for Health and Care Excellence (NICE) guidance,^27^ and is likely to be the case in any larger trial.

The co-participatory intervention development process aimed to ensure the intervention was as effective and engaging as possible, illustrated by the positive participant feedback in the qualitative process analysis (although participants who chose to take part in interviews may have been more engaged and positive about the intervention than those who did not). However, further co-development — particularly with participants representing potentially underserved groups — may improve adherence to the intervention and enhance clinical effectiveness.

The pilot trial has been conducted before the COVID-19 pandemic, which had not only a profound influence on daily clinical practice but also led to a considerable reduction in all-cause ARTI.^28^ Since all restrictions have been lifted for some years and with ARTI incidence and associated antibiotic prescribing on the rise in the post-pandemic era,^29^ we believe that our feasibility outcomes data are applicable to the post-pandemic era as well.

### Comparison with existing literature

To the best of our knowledge, no previous trials of saline nasal irrigation for acute sinusitis have been performed hampering comparison of our findings to other trials of saline nasal irrigation. The benefits of saline nasal irrigation for relieving ARTI symptoms are uncertain,^9,10^ while there might be some benefit of this intervention in patients with chronic sinusitis.^11^ A trial of advice to high-volume nasal irrigation using a neti pot in patients with recurrent or chronic sinusitis demonstrated symptomatic benefits.^12^ Remarkably, most participants were still using the intervention 6 months later and reported they were less inclined to consult a doctor in future episodes and use OTC medication.

### Implications for research and practice

Our pilot trial was not designed and therefore not sufficiently powered to assess clinical effectiveness of our intervention versus usual care in terms of antibiotic consumption, (duration of) symptoms, and costs. While we did observe lower rates of immediate antibiotic prescribing in the saline nasal irrigation group, the low sample size did lead to imbalances of important prognostic factors at baseline and potential selection bias owing to attrition led to unreliable estimates of clinical outcomes during follow-up. We, however, did show that saline nasal irrigation is deemed acceptable for adults with acute sinusitis and that a trial of such intervention in primary care is feasible. A trial with a sufficiently large sample size is required to assess the clinical and cost-effectiveness of our intervention for adults with acute sinusitis.

In conclusion, our randomised controlled pilot trial in UK primary care showed that the advice to use high-volume hypertonic saline nasal irrigation with a delayed antibiotic prescription is deemed acceptable for adults with acute sinusitis and a trial of such intervention in primary care is feasible. A large trial is warranted to assess the clinical and cost-effectiveness of this intervention for this common condition.

## Trial registration number

ISRCTN13373396 (https://doi.org/10.1186/ISRCTN13373396).

## Data

The dataset relied on in this article is available from the corresponding author on reasonable request.

## Public and patient involvement

Samantha Richard-Hall was involved in the discussion about the idea for the study, the development of the protocol, development of the trial materials (patient-facing information) and development of the intervention, and authorship of the article.
